# Maladies Associated with and Dependent upon Affections of the Teeth

**Published:** 1891-05

**Authors:** Richard Grady

**Affiliations:** Baltimore.


AMERICAN JOURNAL

—OF—

DENTAL SCIENCE.

VOL. XXV. BALTIMORE, MAY, 1891. NO. 1.

ARTICLE I.

### MALADIES ASSOCIATED WITH AND DEPENDENT UPON AFFECTIONS OF THE TEETH.*

BY RICHARD GRADY, M. D., D. D. S., OF BALTIMORE.

[Reprinted from the "Maryland Medical Journal."]

There is a field of surgery and pathology, a sort of debatable ground between that occupied by the physician and the dentist, which is open to further research and which will amply repay the labor of investigation. If with any propriety dentistry is classed as a branch of the healing art—whether practiced as a distinct profession or as a specialty of medicine matters not; the difference is that of name merely—its intelligent pursuit has a basis in common with medicine. A competency to treat intelligently any part of the animal economy presupposes a knowledge of the anatomical structure of the part, the relations to surrounding parts, the nervous, arterial and venous connections, the recognition of pathological conditions, and the philosophy of therapeutics. If pathology means perverted nutrition, and if dental lesions are expressions of such perversions, the consideration of their cause and treatment, whether local or constitutional, expresses the medical aspect of the practice.

The physician, the surgeon, the dentist, have necessarily many practical duties in common; a healthy interchange of thought, therefore, would result in profit and instruction alike to all, besides acting as a stimulus for greater and more painstaking scientific inquiry. It is acknowledged on all hands that it is utterly impossible for any one man to master all the details of medical and surgical practice. Few minds can even approach that universality of genius which characterized Hippocrates and John Hunter; hence specialism is the inevitable outcome of the extension of medical art and science. The labors of the rhinologist and the surgeon-dentist often bring them on to a common ground—the region of the antrum of Highmore. The two senses of hearing and seeing being by far the most frequently injured by the reflex neurosis starting from the teeth, the aurist and ophthalmologist must unite with the dental surgeon and he with them, in the advancement of their respective sciences; sciences that have much in common. The recognition of overlapping fields and interlocking realms of study by the neurologist and the dentist will result in the relief of immeasurable suffering, because it is known that the teeth and their pathological states sustain intimate relations with all portions of the body.

The simple lesson emphasized in this paper is one that is hardly recognized at its true value by the general practitioner or the dentist: it is, (1), that a pain in a tooth by no means indicates that the tooth is the source of the trouble; it may be in another tooth, or in other tissues, near or remote; (2), dental disorders may induce pathological conditions in other parts of the body or in the nervous structures themselves without the existence of any subjective intimations of pain in the teeth on the part of the patient. In other words, one may have toothache in the brain, the ear, the stomach; or one may have headache, gastralgia, etc., in the teeth; just as "eye-headaches" are usually referred to the brow, or to other portions of the head.

Eminent surgeons have been scarcely aware how serious are some of the maladies directly dependent upon tooth-disease and how largely the pathology of the teeth is associated with morbid changes in contiguous structures. The fairness of this statement is illustrated by examples of imperfect knowledge in cases which required not the scalpel and the saw, the bone-nippers and the gouge, but ordinary dental instruments. A tooth has, by an error in diagnosis, been mistaken for exostosis of the jaw-bone, resulting in the excision of the angle of the lower jaw, which entirely destroyed mastication. Considerable portions of the jaw-bone, even as much as "half the inferior maxilla," have been removed for dentigerous cysts, operations having been undertaken from an incorrect diagnosis, which a knowledge of the real nature of the disease and an early interference might have rendered quite unnecessary. An impacted tooth, producing a tumor in the right upper jaw and cheek, has been mistaken for cancer; and, in the belief that it was malignant, an operation was begun for its extirpation. The external orifice of an outward-pointing alveolar abscess, dependent on a carious tooth, has been attributed to diseased bone in those conditions in which the opening occurs on the surface of the face, and an easily cured malady has been allowed to run its course unrestrained, and permanently disfigure the face. The symptoms produced in the painful eruption of wisdom teeth have been suspected for scrofulous caries of the jaw, for syphilis and for cancer; and Velpeau, who with other French surgeons has been particularly alive to the importance of these cases, remarked in a lecture that the subject has been "too much neglected by medical men."

General systemic disturbances, resulting from diseased dental organs, might be considered as impossible were it not for the instances with which every dental practitioner is familiar, in which debility, sleeplessness, nervous derangements, mental depression, etc., after resisting constitutional medication, have yielded promptly to such treatment as was found necessary to restore a healthy condition of the mouth. Indeed, so frequently have fretful, nervous, irritable, despondent conditions been found to depend upon dental troubles, it would appear to be a plain duty to have the mouth and teeth carefully examined by a competent practitioner in the event of failure to discover other causes for such manifestations. So many and so varied are the disturbances radiated or reflected to other organs, or dependent upon constitutional irritation from dental affections, their enumeration in detail would be tedious to the reader and their admission to this paper would have swelled it to an inconvenient size; briefly, however, it may be said that the inharmonies thus set up may range from a mere sense of discomfort up to and include the gravest and most formidable derangements, involving even life itself.

*Amaurosis* has been caused by crowding of teeth; has been consequent upon acute abscess of the antrum, produced by a carious tooth; has been caused by a carious molar and a splinter of tooth-pick embedded in the alveolus. *Facial neuralgia* has come from dentine excrescence in the pulp cavity of a tooth; *cranial neuralgia*, from an impacted canine tooth; intense and *general neuralgia* from exostosis on the roots of teeth; *neuralgia of the arm*, from carious teeth and from undue pressure of artificial teeth; *neuralgia of the neck and arm* from a carious molar; *neuralgia of the face, neck and arm*, with *partial paralysis of arm*, from a carious wisdom tooth; intense *nenralgia of the eye-ball and face* and *alteration of the color of the iris*, from carious teeth. *Deafness*, in which the loss of hearing was evidently reflex paralysis, has been caused by a carious tooth, and hearing returned within an hour after the extraction of the tooth, to the patient who had been deaf for four days. An impacted lower tooth (bicuspid), has caused repeated *abscesses under the tongue*, with *thickening of the lower jaw*. Interesting *examples of perverted nutrition*, dependent on dental nervous irritation, are recorded, in which the *tongue* has been *furred on one, and only one, side*, and that side corresponding with carious or painful teeth; in which the *hair of the left temple had turned gray*, the change occurring coincident with and apparently dependent upon, severe neuralgic pain of that side of the head, the painful affection having been caused by a carious molar tooth, *the hair of the right temple remaining black*; and in which *ulceration of the auditory canal*, depending upon a diseased molar tooth in the lower jaw on the same side, healed after the tooth had been extracted. *Superficial sloughing* of the cheek has been caused by a carious "tooth-stump," on whose removal the slough separated, the sore healed, and it never recurred. Tomes gives a graphic account of a terrible *example of fatal lock-jaw*, " induced by pivoting with a gold peg an artificial tooth upon a tooth-fang with a newly exposed raw pulp;" and a similar case is related where *trismus, followed by tetanus and death*, came from the mechanical irritation of the pulp. A very interesting case of *epilepsy* from a carious tooth, conclusive as to its cause, is published; also one of *wry neck*, in which the head of the patient was drawn down nearly to the left shoulder, accompanied with considerable pain. Similar cases could be enumerated almost indefinitely. (The cause of so-called *reflex* troubles and the circles of *sympathies* between the mouth and other portions of the animal system can only be thoroughly understood through a familiarity with the origin, distribution and relation of the nerves, which the writer assumes is possessed by those who will read this paper.)

But the sympathetic or reflex disturbances of harmony caused by dental irritation are not more interesting or instructive than are the *converse* manifestations of pain or discomfort experienced in the teeth, but originating elsewhere. Nor will it be irrelevant to the general subject of this paper, but very worthy of observation, to note that many of the instances of painful affections of the fifth nerve are well marked examples of reflected sensation, the primary irritation being in the stomach or intestinal canal. The pain over the eyes so commonly associated with derangement of digestion, and which may frequently at once be relieved by correcting the acidity of the stomach, is a familiar instance. The condition popularly known as "biliousness," among numerous other manifestations, not seldom reveals itself by a peculiar discomfort produced in the teeth, which, variously described, may be summed up in the phrase "exalted sensibility." An attack of dyspepsia is by many more quickly recognized through disagreeable sensations in the teeth than by any special stomach disturbance. In sea-sickness and in sick headache the nausea is sometimes preceded by intense neuralgia in the teeth and jaws, promptly disappearing if vomiting be induced. In some persons hunger will excite markedly disagreeable sensations in the teeth. A case is published of a gentleman, who, while convalescing from typhoid fever, was seriously annoyed by painful sensations in two of his molars whenever he became hungry. These uncomfortable sensations would rouse him from sleep, and could not be allayed except by the introduction of food into the stomach, when instant relief followed. In another curious case of nervous debility the approach of a thunder-storm or a marked atmospheric variation always produced a most tantalizing sensation of discomfort in the teeth, causing them to feel as though they were denuded of enamel. The singular affection, "brow-ague" (*hemicrania*), which, by yielding to quinia, reveals its malarial origin, is frequently alternated or associated with periodical pain in perfectly sound teeth; sometimes it depends on the irritation of a nerve in a decayed tooth.

It has been noted that a neuralgia originating in a diseased tooth may express itself in the face, eyes, ears, or more remote parts; so by the same methods of radiation or reflection, *reversed*, a neuralgia having a general or constitutional cause, as malaria, or a local cause in the stomach, or elsewhere, may manifest itself in one or more teeth. There is a form of toothache not inappropriately termed "hysteric toothache," which seems to depend upon emotional rather than physical excitants, and is more amenable to mental impressions than to local or general medication. Rheumatism sometimes produces agonizing pain in the jaws, and either by direct influence, by the sympathy of contiguity, or by radiation, may so powerfully affect an individual tooth that the patient can hardly be persuaded that instant and complete relief would not follow its extraction. It is apparent, therefore, that many severe, remote, sympathetic and reflex derangements may be associated with and dependent upon affections of the teeth. It is a sad mistake for the patient, or the physician, or even the dentist, to consider the teeth as mere mechanical organs, requiring only mechanical treatment, and to ignore, therefore, their nervous relations to the entire system.

There is certainly through the period of dentition an increased susceptibility to nervous and digestive troubles. Causes which at other times have no appreciable effect may then be fraught with danger. An exposure to cold, an attack of indigestion, anything which introduces inharmony into the functions of animal life, may result in a disturbance of the processes of dentition. Difficult dentition may, therefore, be charged with causing or aggravating various disorders; as these, on the other hand, may be reasonably suspected of interfering with the natural eruption of the teeth. It is certainly unsafe to ignore the complications possibly due to dentition, if any derangement of the health of the child occur during the period when the teeth are erupting. As a rule, the amount of irritation holds a relation to the number of teeth advancing simultaneously; but, owing to the varying susceptibility of individuals, a single tooth may cause more disturbance in one case than a half-dozen will in another. Manifestations of increased constitutional disturbances are likely to appear in persistent and copious diarrhœa, nausea, high fever, and, not infrequently, convulsions.

There is reason to believe that earache is often associated with, and dependent upon, the difficult eruption of one or more teeth, and that, apart from the aggravation of the fever and the increased liability to convulsions incident to this added anguish, there is also the possibility of the loss of hearing (entailing in young children the loss of speech), from the congestion and inflammation which results. But this is not the only, indeed not the chief, danger; the inflammation is liable to extend to the membranes of the brain and end in death.

The facility with which an irritation, originating in the mouth, may be continued to the ear and thence to the brain, can only be understood by a recognition of the intimate relations which exist, especially in the infant, between the parts concerned and the elaborate nervous connections; but the danger is a real one and should never be lost sight of in the treatment of a child suffering from teething.

Sometimes the irritation of dentition may produce the most serious constitutional derangements without the *local* manifestations, indicated usually by increased redness, swelling and hardness of the gum; and later, by the peculiar whiteness caused by the pressure of the coming tooth. That such may be the case is apparent when the conditions of the parts are understood. The troubles of dentition are caused to some extent by the direct pressure of the advancing tooth, and the consequent irritation of the nerves of the gums; but this is not the only, nor is it believed the principal, factor of disturbance.

It must be borne in mind that at the time of eruption the roots of the teeth are not yet complete; that, instead of the conical termination and minute opening which characterize the root of the perfected tooth, the aperture is quite large and its edges thin and sharp. In estimating, therefore, the mischief which may result because of a lack of accordance between the eruption of a tooth and the absorption of the tissues which impede it, we may imagine the sensitive nerve, which, when exposed to decay, is so intolerant of contact even with the atmospheric air, held between the long socket and the sharp edge of the incomplete root by the backward pressure of the resisting gums, and thus giving rise to a true toothache, comparable only to that exquisite torture which is experienced in after life from an exposed and irritated pulp.

It is not difficult to comprehend that a free division of the gum over the tooth or teeth thus situated may, by removal of the pressure, give immediate and complete relief. The objections against lancing find answer in the immediate, manifest,and complete relief to the infant which so often follows the operation that the relation of cause and effect is apparent to any observer; also, in the testimony of every adult who has experienced the comfort resulting from the employment of the lancet in the case of the eruption of wisdom teeth with difficulty. Only an ignorance of the anatomy of the mouth, entirely inexcusable in any physician or dentist, could lead to the infliction of permanent injury.

After an exhaustive examination of medical and dental literature, from hundreds of cases that might have been cited or referred to, there are included in this paper only those of more than ordinary interest and usefulness. In this connection it is worthy of note that ophthalmologists hardly entertain the proposition that pathological conditions of the teeth may by the intervention of the nervous system result in ocular disorders. The matter is not even alluded to by some authors, while others do not more than refer to it. Only a small number of clinical cases is to be found in ophthalmological literature. The subject has been almost ignored by medical men; a praiseworthy exception being Dr. George Reuling, professor of ophthalmology and otology in the Baltimore Medical College, who, pointing to the fact above stated, solicited from the dental profession of Baltimore illustrative cases that had come under notice.

Passing, after mentioning simply the pathological conditions connected with the teeth which most frequently give rise to pain—caries, exposed pulp, congested and inflamed pulp, dead and putrescent pulp, nodular dentine in the pulp, inflammation of peridental membrane, fracture of the crown—to reflex odontalgia, it may be remarked that the term, "sympathy," used by dental writers to express the sensation of pain in one tooth near or remote from the seat of disease, is wholly without significance. That an irritation of the nervous connection of one tooth may set up an odontalgia that the patient locates in another tooth, whilst the inciting tooth may be painful or not, is a fact long recognized. Friedberg [1] alludes to a case where a patient suffering for twelve days begged to have the second and third molars of the right side extracted. Both were sound, but an inner incisor was carious, though indolent to percussion; this was extracted and the odontalgia at once ceased. Dr. Brunton [2] cites the case of a family servant who had been suffering from a very severe temporal headache, and at the same time a severe toothache. He did nothing for the headache, but applied a pledget of cotton soaked in carbolic acid to the suffering tooth. Finally, word came to him that the girl had changed the pledget of cotton from the decayed molar that seemed to her the aching one to another hollow but painless tooth, when both the headache and the toothache vanished as if by magic.

That there have not come to hand more quotable cases illustrating neurosis of ocular and nasal origin may be because physicians have not been sufficiently and accurately observant. A case is related in which a lady suffered extremely with toothache for several days after undergoing a slight operation in the nasal chamber. Dr.

Galezowski[3] cites a case where *iritis*, with external inflammation of the eye, had existed for fifteen days, coexisting with great dental neuralgia on the side of the affected eye. J. Hutchinson[4] publishes the following case: "A young man, the subject of acute ulcers of the cornea from injury, with hypopyon, chemosis, and much pain, complained that his eye made his teeth and ear ache: had never heard it mentioned so definitely before. Here we have an instance in which a pain certainly beginning peripherally induced pain in two other distinct and somewhat distant peripheral parts."

Of the peripheral sources of reflex odontalgia, by far the most generally admitted and best illustrated are those proceeding from the disorders of the viscera of the abdominal cavity. Gynecologists have often noticed that toothache is frequently associated with uterine affections, and the general practitioner, as well as the dentist, has more frequently found it to be bound up with disorders of the alimentary canal. Dr. Pierce[5] has communicated a case in his practice where a man, who had never been troubled with neuralgia, suffered for a week with severe pains in his face, thongh more particularly in his teeth. There was no condition of the teeth that would account for the severe pain. The patient's bowels had not been opened for a week or more. Free purgation gave complete relief. Dr. J. W. White,[8] in an article upon the "Systemic Causes of Odontalgia," alludes to the influences of the alimentary canal and bladder in the production of pain in the teeth. That morbid conditions of the uterus in the pregnant and non-pregnant state very commonly cause odontalgia, both in diseased and sound teeth, is a fact which has long attracted the attention of dentists and obstetricians. Dr. Garretson[6] relates the case of a woman who complained of odontalgia, which had persisted for nine weeks. A carious tooth was removed without any apparent benefit. In the absence of any lesion in the mouth to account for the pain, a general examination of the system was instituted, when it was discovered that the inner surface of the fundus of the uterus was ulcerated. Its cure was soon followed by a disappearance of the toothache. Dr. Storer[7] relates two cases of neuralgia of the dental and ginglval nerves occuring during the pregnant state.

That thrombi, tumors, inflammatory processes, etc., at the base of the brain, within its substance, or about the cortex, may produce odontalgia, is a proposition which finds but little support in the records of clinical cases. Nevertheless, such instances exist. Coleman[9] mentions the case of an insane lady who repeatedly troubled him and other practitioners "to remove her sound teeth on account of the uncomfortable sensations which she referred to them." Dr. Stellwagon[10] also states that he gave great offense to a military officer by refusing to extract some perfectly sound teeth, which were the seat of severe odontalgic pain. The patient shortly after began to exhibit symptoms of softening of the brain. Rosenthal[11] states that he has "seen old men, sixty to seventy years of age, suffering from melancholia complicated with neuralgia of the dental branches; these cases must be attributed to senile changes in the tissues (osseous canals or arteries)."

It is quite certain that hysteria frequently gives rise to or is associated with odontalgia. Dr. Richardson[12] has stated that it is more common than generally supposed, and relates a case in illustration of his view that the toothache of pregnancy is frequently, if not always, connected with hysteria.

It has long been known that the various systemic conditions caused by the poisons of malaria, gout, syphilis, etc., while having no special and remarkable tendency to produce pain which the patient localizes in the teeth, are yet frequently provocative of severe and obstinate forms of odontalgia. There can be no doubt that these kinds of reflexes exist, as noteworthy cases illustrate. In malarial districts, toothaches of a distinctly periodical character are observed. In the gouty and rheumatic diathesis the pain which usually localizes itself in the joints not infrequently selects one or more teeth for its local expression. In such cases the pain assumes the specific character of these diseases. Dr. Flagg [13] reports a case of odontalgia of malarial origin, in which the patient had been under treatment for pain in her face for eighteen months. Dr. Latimer [14] mentions a case of odontalgia of gouty origin, in which a violent toothache was suddenly exchanged for a gouty pain in one of the great toes; and Dr. Harris [15] reports a case in which a victim of gout for fifteen years went to him for odontalgia. An example of odontalgia of specific origin is given by Dr. Pierce [5] of a young lady who had been suffering for several days from severe pain in all the teeth, though they were apparently free from disease. A few days later the trouble entirely disappeared upon the appearance of the eruption of measles.

Having briefly passed in review cases of local and systemic conditions which are reflected *to* the teeth,giving rise to odontalgia, it only remains, in conclusion, to note some cases in which affections of neighboring and distant organs are set up by pathological conditions of the teeth as primary inciting causes. *Why* the irritation from morbid conditions of the teeth should in one case result in wryneck or facial spasm, and in another in blindness, in deafness, in chorea, in dyspepsia, in epilepsy, or in mania, is an inquiry beyond the purpose of this paper. But in this connection there is one thing beyond question : the cases presented leave no room for doubt that morbid conditions of the teeth may be, in other organs far removed, the fruitful sources of troubles whose real origin was hardly to be suspected.

No anatomical or scientific order has been attempted in arranging the following illustrative cases; those of external and noticeable functional disorders will be taken first.

Mr. Hancock [16] relates a case of divergent strabismus and ptosis; the left eye was closed; two carious molar teeth in the left side of the upper jaw were extracted; in four days the ptosis was cured and the strabismus so slight as not to require an operation; three weeks later the patient was discharged cured. Dr. Ely [17] reports three cases of paresis of the ocular muscles, which are particularly instructive, as the relation of cause and effect between the dental irritation and the ocular defects seems to have been thoroughly established. Mr. S. J. Hutchinson [18] relates a case of a lady who suffered several months from a spasm of eye-lid; the removal of teeth was attended with satisfactory results. Dr. Keyser [19] reports a case of corneal inflammation and beginning ulceration; on the extraction of the tooth and ordinary treatment, the cornea became clear again. To test the failure or loss of accommodation of the eye, Hermann Schmidt [20] examined ninety-two persons suffering from caries of the teeth, periostitis, neuralgia, etc; only nineteen had a normal accommodating power, and seventy-three were decidedly deficient. The relation of toothache and dental irritation to glaucoma simplex has been studied by Mr. Priestly Smith, [21] who found a distinct difference in the tension in the two eyes in six cases out of sixteen.

It is now pretty generally admitted that amaurosis often results from the irritation reflected from diseased teeth, and almost wholly disappears after their extraction. A striking illustration of the close relationship of amaurosis and dental irritation is reported by Dr. De Witt, [22] in which amaurosis of right eye of twelve years' duration was cured by the extraction of the tooth. Many other remarkable cases of amaurosis have been reported by Keyser, [19] Hutchinson, [30] Hunter, [23] Galezowski, [24] Abbott [25] Alexander, [26] Salter. [27]

That aural troubles are excited through reflex action from irritation of dental nerves is a truth no longer questioned. The fact has become so apparent as to be well recognized by aural surgeons. Thus out of eighty infants under fourteen months of age, examined by Dr. Wreden, [28]

of St. Petersburg, more than eighty per cent. had some form of ear trouble. Sexton,[29] in reviewing his records of some 1500 cases, says, "perhaps one-third owe their origin or continuance to diseases of the teeth." The same writer has also called attention to the occurrence of otalgia and other forms of aural trouble as due to the irritation of carious and diseased teeth, and more particularly of pulpless teeth. For further information upon this subject, so important in all its bearing to both physician and dentist, see "Medical Record" for 1884 and "Dental Cosmos" for 1885.

Neurosis, peculiar to the alimentary canal, larynx, heart and even uterus, arising as a sequence to dental irritation, has from time to time been observed by clinicians. That dental irritation should be reflected to these organs might at first glance seem remarkable, but it is not more so than that mental emotions should result in inhibition of gastric juice or that ear lesions should cause laryngeal cough. A few cases of decided interest and suggestiveness have been recorded. Dr. Masterman[16] reports a case of obstinate vomiting from irritation of the alveolar and lingual nerves from the deposition of tartar. Valleix[31] records a case of nausea, vomiting, convulsions and death from painful dentition. Dr. Paasch[32] records the details of a case of laryngeal cough caused by the eruption of teeth. Dr. Pointis[33] records the case of a patient who, after an attack of the toothache, "suddenly lost his voice;" the aphonia was followed by anorexia, cough, wasting and feverishness. Dr. Hullihen[34] reports a case of vicarious menstruation from diseased teeth in a young lady, aged seventeen.

That the eruption of the teeth and the development of disease of the hip joint should in any way be associated as cause and effect may appear highly improbable. Yet such a relation has been asserted; and Dr. Mulreany[35] calls attention to the fact that, in scrofulous children, hip disease makes its appearance during the eruption of the molar teeth and often passes away after they have pierced the gum. In two cases which he relates he gives it as his opinion that dental irritation was the direct exciting cause.

It seems to have been thoroughly established by the recorded observations of clinicians that dental irritation may give rise to neuralgia. Neucort[36] early emphasized the part played by the teeth and sought to lay down rules for diagnosing a neuralgia of dental origin; and detailed seventeen cases of neuralgia of dental origin, illustrating the rules. Friedburg has reported in detail four cases, the first being a case of one-sided facial neuralgia of five years' duration, cured immediately by the extraction of two carious teeth. Many interesting cases of facial neuralgia, incited by dental lesions will be found in Dental Cosmos, 1874.

Reflex paralysis has frequently been observed as the result of diseases of the alimentary canal and urinary organs. Similar reflex paralyses involving the face, arms and legs induced by dental irritation are recorded. Gillman[37] records a case of facial paralysis from a carious tooth; so do Bacon, McQuillen, Nairne. Dr. Whitney[38] relates a case of partial paralysis of the arm, consequent upon caries of the wisdom tooth. Levison records a remarkable case of general paralysis from carious wisdom teeth. Dr. Fliess[39] ascribes infantile paralysis to teething; and Dr. Brown-Sequard[40] says: "Usually, enteritis in teething is produced by a reflex action, and paralysis in the same way."

It has long been known by ophthalmologists that errors of refraction and abnormal ocular conditions are fruitful sources of headache. Diseases of the teeth are certainly next in importance to those of the eyes in producing this troublesome affection. In a general way, almost all writers have recognized the teeth as one of the causes of headache. Dr. Brunton, who is quoted elsewhere, having verified the fact that there may be headache caused by dental disorders without any tooth-ache, has made it his rule, in all cases of headache, first, to examine carefully the teeth. Dr. Baxter records a case of persistent headache the result of exostosis.

That dental inflammation and disorders are often provocative of epileptic attacks appears quite certain from the following cases, and also from the character of the cause and effect. Dr. Tomes [41] relates the case of a lad admitted to the hospital for epilepsy, with whom the usual remedies were tried for six weeks without effect. Decayed teeth, of which he did not complain, were removed. "During the eighteen months that succeeded the removal of the diseased teeth he had not suffered from a single fit, though for many weeks previous to the operation he had two or three per day." Trousseau [42] relates the case of a patient who had been subject to monthly attacks of epilepsy for several years. Many remedies had been tried in vain. From the day that some carious teeth which ached constantly were extracted, the fits disappeared. Dr. Booth [43] relates a case of eleptoid convulsions of right arm from carious teeth, occurring in an unmarried woman. Similar cases, depending for their cause upon diseased teeth and completely cured after their removal, have been reported by Drs. Castle, Waite, Field and Rush.

In hysteria, as in many other diseases, morbid conditions of the teeth not infrequently act as exciting causes of the paroxysms. Dr. Lederer [44] reports a case of a strong, healthy girl who had by his advice an artificial tooth pivoted. Soon afterward the girl was seized with hysterical attacks and convulsions that broke her health and kept her in bed most of the day. At this time the patient was absent, but the doctor, hearing that she was suffering from hysteria, had his suspicions aroused, and (after many other physicians had been called in and failed to give relief) he ordered the artificial tooth taken out, and as if by magic every symptom of hysteria and paroxysmal attacks disappeared immediately, and only reappeared when later there was another attempt made to wear the tooth again. Dr. Richardson mentions a case of a young girl who came under his care for hysteria of an eleptiform character. Various remedies had been tried without any benefit. Finally, after great pain, she erupted a wisdom tooth, after which she lost all her hysteric symptoms. He never meets with a case of hysteria now, "if the excitant seems to be local, without asking in the most solicitous manner after the wisdom teeth."

In but few of the standard works on nervous diseases is the eruption of the teeth ever associated with the appearance of choreic movements. Eulenberg, "on several occasions found the disease, when due to dental disorders, disappear after the extraction of the carious tooth and again make its appearance" in fresh troubles of teeth.

The occurrence of insanity as a result of the pain and irritation caused by the eruption of the teeth was first noticed by Esquirol. Dr. Maudsley in commenting upon the relation of morbid bodily states to disordered mental functions says: "I have under observation now a lady who suffered from an intense neuralgia of the left half of the face; after the removal of a tooth suspected to be at the root of the mischief the pain ceased, but an attack of melancholia immediately followed." Mr. D. Corbett relates a case of insanity from overcrowding of the teeth, a girl of thirteen years who would "run about the room biting at chairs, tables and door-handles. In the street she would run off from her attendants and attempt to bite the lamp-posts." The biting propensity completely disappeared on the removal of two teeth. Dr. Tyler relates a case of mania from carious teeth, in which a young lady had several decayed teeth removed, remaining "under the influence of ether which was given at the operation for twenty-four hours; after that she was cured of her mania." Dr. Pepper relates an interesting case of insanity caused by the irritation of carious teeth, on whose removal the patient was cured. The trouble again returned after three weeks, when a portion of the inferior dental nerve (about half an inch) was removed and the patient was permanently cured.

REFERENCES TO ILLUSTRATIVE CASES CITED.

1. Virchow's Archiv. Band xviii.
2. St. Barthol. Hosp. Reports, Vol. xix.
3. Jour. d'Ophthalmologie, tome i.
4. London Medical Mirror, 1869.
5. American System of Dentistry, Vol. iii.
6. System of Oral Surgery, 4th ed.
7. Atlanta Med. and Surg. Journal, 1859.
8. Dental Cosmos, 1872.
9. Dental Surgery and Pathology.
10. Ibid.
11. Diseases of Nervous System.
12. Diseases of the Teeth.
13. Dental Cosmos, Vol. iv.
14. Dental Cosmos, 1865.
15. Dental Surgery.
16. Lancet, 1859.
17. N. Y. Medical Recork, 1882.
18. Med. Surg. Rep., 1885.
19. Dental Times, 1870.
20. Archiv. fur Ophthalmologie, xiv.
21. Glaucoma, its Causes, etc., 1879.
22. American Journal Medical Sciences, 1868.
23. American Journal Medical Science, 1841.
24. Archives generales de Medicine, tome xxiii.
25. Dental Cosmos, 1870
26. Klin. Monatbl fur Angenheilk, 1868.
27. Lancet, 1862.
28. Woakes "Deafness, Giddiness, etc., 1879."
29. American Journal Medical Science, 1880.
30. Ophthalmic Hosp. Reports, 1885.
31. Clinique des Hospitaux des Enfans, 1846.
32. Journal fur Kinderkrank, 1856.
33. Journal des Conn. Med. prat., 1846.
34. American Journal Dental Science.
35. N. Y. Medical Journal, 1874.
36. Archives generales 1849, 1853.
37. Boston Medical and Surgical Journal, 1867.
38. Buffalo Medical and Surgical Journal, 1865.
39. Journal fur Kinderkrank, 1849.
40. Lancet, 1860.
41. System of Dental Surgery.
42. New Sydenham Soc., Vol. i.
43. American Journal Medical Science, 1870.
44. Wiener Medizinische Presse, 1866.

